# New Insights into the Chemical Profile and Health-Promoting Effects of Mushroom *Sarcodon aspratus*

**DOI:** 10.3390/foods15213861

**Published:** 2026-10-28

**Authors:** Nilakshi Dhanushika, Baojun Xu

**Affiliations:** 1 Food Science and Technology Program, Department of Life Sciences, Beijing Normal-Hong Kong Baptist University, 2000 Jintong Road, Tangjiawan, Zhuhai 519087, China; ag19026@agri.pdn.ac.lk; 2 Faculty of Agriculture, University of Peradeniya, Peradeniya 20400, Sri Lanka

**Keywords:** *Sarcodon aspratus*, *β*-glucan, phytochemical profile, immunomodulation, gut microbiota, antioxidant, ergosterol peroxide, functional food

## Abstract

* Sarcodon aspratus* (Berk.) S. Ito is a traditionally prized wild edible mushroom whose primary and secondary metabolites, particularly complex β-glucans and ergosterol peroxide, have been associated with antioxidative, immunomodulating, anti-inflammatory, and gut-microbiota-related effects in experimental models. While its culinary value and ethnomycological history are well established in East Asian traditions, the molecular mechanisms underlying these effects remain incompletely characterized. Water-soluble β-glucan fractions reported from *S. aspratus* include high-molecular-weight fractions of approximately 4.3 × 10^5^ to 6.7 × 10^5^ Da (about 430–670 kDa), although reported composition and activity vary with extraction and purification procedures. Experimental studies suggest that polysaccharide structure—including molecular weight, branching, linkage pattern, purity, and solubility—can influence receptor interactions and downstream immune responses. Ergosterol peroxide and L-pipecolic acid have also shown apoptosis-inducing and ACE-inhibitory activities, respectively, in experimental systems. However, most evidence remains limited to in vitro and animal studies, and species-specific chemical profiling, standardized extraction procedures, bioavailability studies, and human clinical trials remain important knowledge gaps. Future research should therefore focus on mechanistic validation, standardized characterization, safety assessment, and sustainable cultivation before the health-promoting potential of *S. aspratus* can be translated into standardized functional foods or nutraceutical products.

## 1. Introduction

### 1.1. Taxonomy and Classification

**Figure 1:**
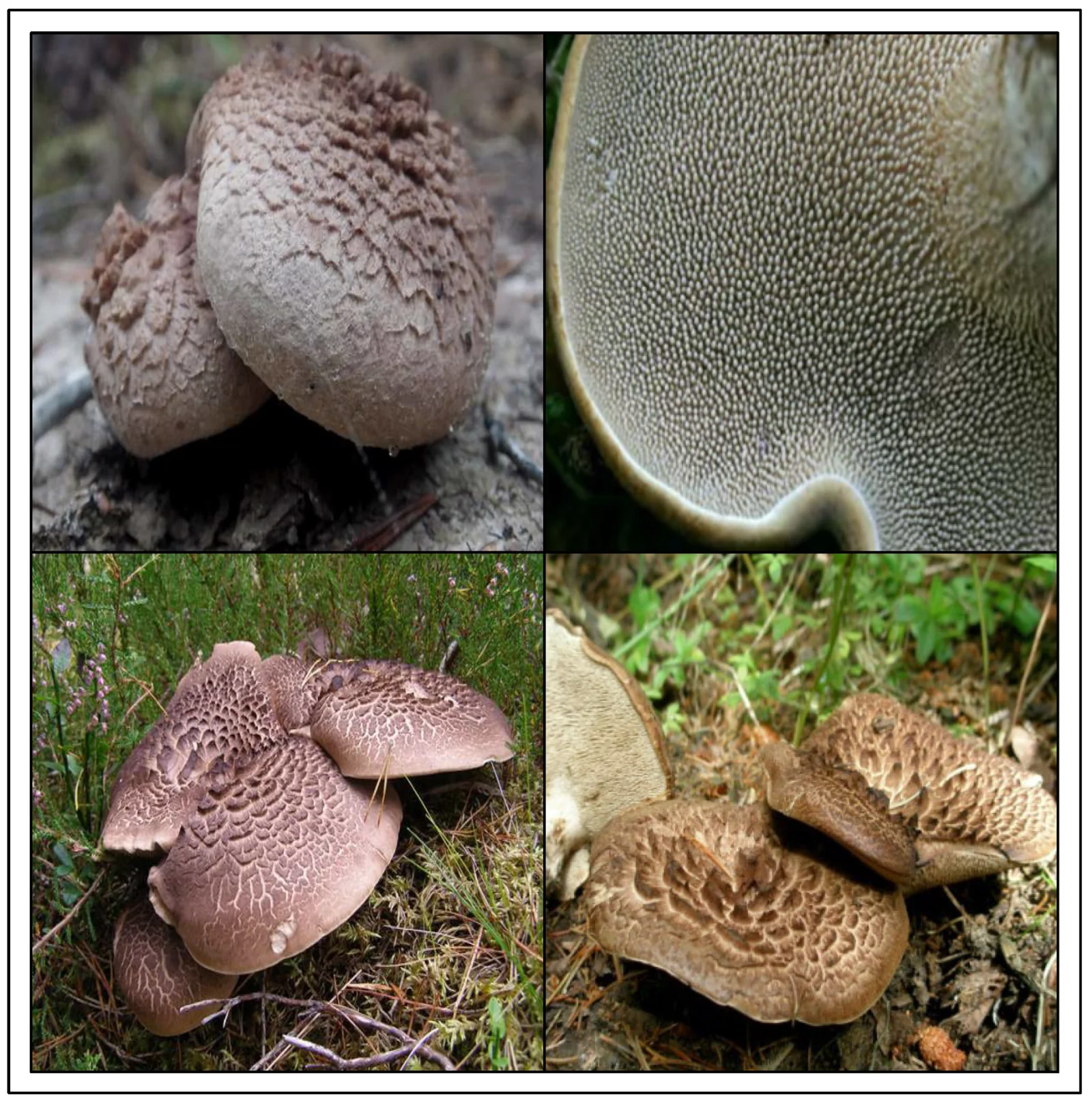
Morphological characteristics of *Sarcodon* species showing the scaly cap surface and the distinctive tooth-like hymenium on the underside of the fruiting body. These spines serve as the spore-producing surface characteristics of hydnoid fungi in the family Bankeraceae [2,3].

*Sarcodon aspratus* (Berk.) S. Ito belongs to the family Bankeraceae, genus *Sarcodon*, and order Thelephorales (Basidiomycota) of basidiomycetes. As a defining morphological characteristic of this lineage of ectomycorrhizal mushrooms, Sarcodon members are hydnoid fungi defined by downward-pointing spines (or “teeth”) on the hymenophore rather than gills or pores. Although taxonomic research on *Sarcodon* has identified a number of closely related species and caused some nomenclatural confusion (for example, between *Sarcodon* taxa like *S. imbricatus* and *S. squamosus*), morphological and molecular investigations confirm *S. aspratus*’s unique position within this group of tooth fungi [1]. The key morphological features of these tooth fungi are illustrated Figure 1.

### 1.2. Geographic Distribution

Most reports of *S. aspratus* come from East Asia, specifically from the wooded areas of China, Japan, and Korea. There, it grows in mixed and montane forests, frequently in ectomycorrhizal relationship with trees. Its distribution matches the ranges of suitable host trees and forest ecosystems in the Northern Hemisphere, as evidenced by the fact that its fruiting bodies usually occur in temperate settings. Studies and herbarium records indicate that *S. aspratus* is a well-established fungal component of the forest myconiota in East Asia, despite the fact that its global distribution is still not fully documented [4].

### 1.3. Traditional Culinary and Medicinal Uses

*S. aspratus* has long been known in East Asian ethnomycology as a common wild edible fungus and functional food. It is especially prized in China, Korea, and the surrounding areas for its flavor, nutritional content, and customary use in regional cuisines. Its extensive use and culinary appreciation in traditional meals throughout different cultures can be attributed to its description in nutritional and biochemical research as a “popular edible fungus” that is both delicious and rich in protein, carbs, and minerals. In addition to being used as food, *S. aspratus* has been used in traditional Asian folk medicine practices. In these practices, the mushroom has been prepared to support digestive health, overall wellbeing, and other traditional health purposes. This has prompted scientific research into the mushroom’s bioactive compounds and possible health benefits. While many of these ethnomedical uses remain anecdotal, they have provided a historical context that has stimulated modern research into the mushroom’s functional constituents and biological activities [5].

### 1.4. Rationale for Reviewing New Chemical and Biological Findings

Scientific research on the chemical makeup and biological activity of *S. aspratus* has only intensified in recent decades, despite its long history of use and culinary value. New research has found a variety of classes of bioactive substances, such as lipid constituents like ergosterol peroxide that induce apoptosis, small molecules like I-pipecolic acid that inhibit enzymes, and polysaccharides that have immunomodulatory, antioxidant, and protective effects. Polysaccharide fractions from *S. aspratus*, for instance, have been demonstrated to prevent oxidative stress and stomach ulcers in animal models by modulating macrophage activity through Toll-like receptor 4 (TLR4)-mediated signaling [5]. Ergosterol peroxide has also been demonstrated to cause leukemia cells to undergo apoptosis, while I-pipecolic acid, which was extracted from the mushroom, shows in vitro suppression of the angiotensin I-converting enzyme, indicating possible antihypertensive benefits [6].

All these results point to a rich chemical profile with intriguing biological roles that go far beyond conventional culinary uses. There is currently no thorough synthesis that incorporates the chemical diversity, mechanistic insights, and possible health advantages of *S. aspratus* constituents, despite the fact that different research has revealed distinct bioactivities and compound classes. Therefore, a thorough evaluation is required to compile existing information, evaluate bioactivity data critically, and direct future studies and therapeutic investigations.

## 2. Phytochemical/Chemical Profile

### 2.1. Primary Metabolites

#### 2.1.1. Proteins and Amino Acid Composition

Proteins from *S. aspratus* have a notable amino acid profile and are nutrient-dense. According to one study, the total amount of amino acids was 98.88 mg/g of necessary amino acids and 65.49 mg/g of non-essential amino acids. This suggests that the amino acid composition is balanced for nutritional quality. Additionally, significant levels of major minerals like Mg, Fe, Zn, and Mn were detected, confirming the nutritional importance of this species.

#### 2.1.2. Polysaccharides

One of the most researched main metabolites in *S. aspratus* is polysaccharides, which are often recovered by hot water extraction and then ethanol precipitation. Using HPSEC, methylation analysis, FT-IR, and NMR spectroscopy, for instance, water-soluble glucan fractions (such as HBP and HCP) with high sugar concentrations and molecular weights between ~4.3 × 10^5^ and ~6.7 × 10^5^ Da have been separated and structurally described. These polysaccharides have demonstrated immunomodulatory activity in vitro and frequently contain *β*-linked glucopyranosyl residues [7].

#### 2.1.3. Fatty Acids

Comparative studies of closely related *Sarcodon* species (e.g., *Sarcodon imbricatus*) show extensive fatty acid variability, including unsaturated fatty acids like oleic and α-linolenic acids, despite the lack of well-documented fatty acid profiles for *S. aspratus.* GC-MS analysis in lipid fractions is usually used to identify these substances. Although detailed fatty acid profiling for *S. aspratus* itself remains limited, studies on wild edible mushrooms including closely related basidiomycete species demonstrate that unsaturated fatty acids such as linoleic (C18:2) and oleic acid (C18:1) typically predominate in mushroom lipid profiles when analyzed by GC-MS and gas chromatography [8].

#### 2.1.4. Minerals and Vitamins

Comparative studies of closely related *Sarcodon* species (e.g., *Sarcodon imbricatus*) show extensive fatty acid variability, including unsaturated fatty acids such as oleic and α-linolenic acids. However, a detailed GC-MS fatty acid profile specific to *S*. *aspratus* remains limited in the available literature. Therefore, observations from closely related wild edible basidiomycetes are presented here as chemotaxonomic context rather than as direct species-specific measurements. Linoleic acid (C18:2) and oleic acid (C18:1) commonly predominate in mushroom lipid profiles analyzed by GC-MS [8]. Dedicated GC-MS profiling of *S. aspratus* is needed to establish its species-specific fatty acid composition [9].

### 2.2. Secondary Metabolites

#### 2.2.1. Terpenoids

*Sarcodon* species are known to produce a variety of terpene-type chemicals, particularly cyathane diterpenoids and *p*-terphenyl derivatives, which support biological functions like antibacterial and antioxidant actions. These chemicals have been identified via chromatographic separation and NMR characterization across several *Sarcodon* species, despite the paucity of through investigations specifically focused on *S. aspratus* [10].

#### 2.2.2. Phenolic Compounds

Direct analyses of phenolic compounds in *S. aspratus* are scarce. Studies of related *Sarcodon* species, including *Sarcodon imbricatus*, and other wild edible mushrooms have reported measurable phenolic contents using spectrophotometric and chromatographic methods such as HPLC-UV. For example, *S. imbricatus* exhibits phenolic compounds associated with radical-scavenging activity [11]. These findings provide useful chemotaxonomic context, but they should not be interpreted as direct quantitative evidence for *S. aspratus*. Species-specific HPLC or LC-MS profiling is therefore required to establish the phenolic composition and its relationship with antioxidant activity in *S. aspratus*.

While direct analyses of phenolics in *S. aspratus* are scarce, studies on *Sarcodon imbricatus* and other wild edible mushrooms have shown significant levels of phenolic compounds measured by spectrophotometric and chromatographic methods such as HPLC-UV, which correlate with antioxidant activity. For example, *S. imbricatus* exhibits high total phenolic contents that are linked to strong radical scavenging effects in antioxidant assays [11].

#### 2.2.3. Sterols

From lipophilic extracts of *S. aspratus*, sterol compounds, in particular ergosterol peroxide, have been identified. Using NMR and MS methods, ergosterol peroxide was discovered and has been demonstrated to have the ability to induce apoptosis in leukemia cells [12].

#### 2.2.4. Unique or Newly Identified Compounds

Using preparative HPLC and chromatographic purification, new cerebroside compounds from *S. aspratus* have been discovered, illustrating the structural diversity of uncommon lipid-derived metabolites in this species. The chemical distinctness of this species is further influenced by other non-volatile natural compounds, such as p-terphenyls and cyathane derivatives [10]. A summary of the main primary and secondary metabolites, their structural characteristics, and their biological relevance in *S. aspratus* is presented in Table 1.

## 3. Bioactive Polysaccharides

In *Sarcodon aspratus*, polysaccharides are among the bioactive chemical classes that have been studied the most. They are known to have a variety of biological functions, including immunomodulation, antioxidant properties, and possible defense against oxidative damage and inflammation. The structural characteristics, molecular weight, glucan content, and structure–activity interactions that have been documented in the literature are compiled in the next subsections.

### 3.1. Structural Characteristics

The majority of the polysaccharides that are recovered from *S. aspratus* are hetero-polysaccharides made up of glucose units with different branching patterns. FT-IR spectroscopy, GC-MS of monosaccharide compositions, and NMR spectroscopy (^1^H & ^13^C) have all been used in structural investigations to show that the primary backbone of these polysaccharides frequently consists of β-linked glucopyranosyl residues with branching at 0–6 locations. O-acetyl and glycosidic connections are examples of functional groups that add to these macromolecules’ conformational variety, which is assumed to affect their biological activity.

According to research employing methylation analysis and NMR techniques, *S. aspratus* polysaccharides are classified as β-glucans with extra sugar chains because they mostly comprise β-(1 → 3) and β-(1 → 6) glycosidic connections. Other mushroom polysaccharides linked to immunostimulatory actions have these characteristics [5,13].

### 3.2. Molecular Weight

The molecular weight (Mw) of mushroom polysaccharides is an important structural characteristic that influences their physicochemical properties and biological activity. In *S. aspratus*, water-soluble polysaccharide fractions obtained by hot-water extraction and ethanol precipitation have been characterized by high-performance size-exclusion chromatography (HPSEC). The reported high-molecular-weight fractions HBP and HCP have molecular weights of approximately 4.3 × 10^5^ Da and 6.7 × 10^5^ Da, respectively (about 430–670 kDa) [7]. These values should be distinguished from lower-molecular-weight β-glucans reported in other mushroom systems. The biological contribution of molecular weight is also dependent on branching, linkage pattern, purity, and solubility; therefore, molecular weight alone should not be considered a sufficient predictor of activity.

Higher-molecular-weight β-glucans may present multiple carbohydrate-binding sites within a single polymer and thereby facilitate multivalent interactions with immune-cell receptors. This can promote receptor clustering and strengthen downstream signaling, whereas smaller oligomers may have weaker receptor cross-linking capacity. However, the relationship is not universal: molecular weight interacts with polymer conformation, branching, solubility, and purity, and comparisons among studies are complicated by differences in extraction and fractionation procedures.

Enhanced biological activity in immune regulation and free radical scavenging is typically linked to higher molecular weights; however, the precise role of Mw in function might differ depending on structural branching and solubility [5].

### 3.3. β-Glucan Content

A well-known subclass of mushroom polysaccharides with immunomodulatory and health-promoting properties is called β-glucans. After extraction and purification, β-glucans in *S. aspratus* are identified with certain enzymatic tests and spectrophotometric techniques. These polymers have β-(1 → 3) main chains with β-(1 → 6) branches, which are structural motifs linked to immune receptor recognition, including Dectin-1 and macrophage Toll-like receptors.

Research has shown that *S. aspratus* extracts contain large amounts of β-glucan, but the precise quantities vary depending on the extraction technique and sample origin. The reported immunostimulatory effects in animal models of inflammation and macrophage systems, including activation of cytokine production, are mostly attributed to the β-glucan concentration [5,14].

### 3.4. Structure-Activity Relationship

• The structure–activity relationship (SAR) of mushroom β-glucans is determined by several interrelated physicochemical features, including molecular weight, backbone length, glycosidic linkage pattern, degree of branching, purity, and solubility. In *S. aspratus*, the reported polysaccharides commonly contain β-(1 → 3) backbones with β-(1 → 6) branches [7]. These structural features can influence how polysaccharides interact with pattern-recognition receptors and consequently affect the magnitude and type of cellular response. Dectin-1 recognizes β-(1 → 3)-linked glucan structures, while TLR-associated responses can contribute to downstream MAPK and NF-κB signaling in immune cells. Importantly, the available evidence does not establish a single structural feature as sufficient to explain all biological effects. Instead, molecular size, branching, linkage pattern, solubility, and purity should be considered together when interpreting the activity of *S. aspratus* polysaccharide fractions.
• Dectin-1: Stimulates phagocytosis and drives the production of key cytokines (TNF α, IL-2, IL-10, IL-12) [15].
• Toll-like Receptors (TLR2/4): TLR-associated signaling can activate MAPK cascades and NF-κB nuclear translocation, contributing to innate immune responses [15].
• Complement Receptor Type 3 (CR3) & Scavenger Receptors: These receptors can contribute to leukocyte adhesion and downstream signaling, although their specific contribution to *S. aspratus* polysaccharide activity requires further species-specific investigation [15].

### 3.5. Molecular Weight and Immune Activation

Molecular weight can influence β-glucan activity because larger polymers may provide multiple receptor-interacting sites and facilitate multivalent receptor clustering on immune-cell membranes. For β-glucans in the approximately ≥100–200 kDa range, this multivalency can favor clustering of receptors such as Dectin-1 and, where relevant, TLR-associated signaling components, thereby facilitating recruitment of intracellular signaling proteins such as Syk and MyD88 and activation of downstream NF-κB and MAPK pathways. In contrast, low-molecular-weight oligomers may have reduced capacity for receptor cross-linking and can compete with larger ligands under some experimental conditions [16]. Nevertheless, molecular weight should be interpreted together with branching, linkage pattern, conformation, purity, and solubility rather than as an independent determinant of activity.

### 3.6. Glycosidic Linkage Type and Receptor Interaction

The specific arrangement of glycosidic linkages is critical for the recognition of -glucans by pattern-recognition receptors, such as Dectin-1 [11]. Interaction with these receptors specifically requires the presence of -(1 → 3) linkages [17]. Studies have confirmed that Dectin-1 binding is exclusive to linked glucose oligomers, with a minimum backbone length—typically between seven and eleven units—required for successful binding and subsequent induction of inflammatory signaling pathways [17]. This specificity ensures that the innate immune system can effectively distinguish these fungal and bacterial markers from other types of carbohydrates.

### 3.7. Degree of Branching and Recognition

Branching at β-(1 → 6) positions can alter the three-dimensional presentation of β-glucan chains and thereby influence receptor accessibility and binding. The β-(1 → 3) backbone provides an important structural determinant for Dectin-1 recognition, while β-(1 → 6) branches can modify ligand conformation and receptor engagement [17]. Thus, differences in branching frequency and branch length may contribute to differences in immunomodulatory activity among polysaccharide preparations. However, direct structure–activity comparisons using purified *S. aspratus* fractions remain limited, so broader mushroom β-glucan findings should be interpreted cautiously [18].

### 3.8. Purity and Solubility

Purity and solubility are additional determinants of β-glucan activity. Highly purified and soluble fractions can provide more reproducible receptor exposure than crude preparations, whose biological effects may be influenced by proteins, phenolics, lipids, or other co-extracted components [10,13]. Solubility also affects the accessibility of polysaccharide chains to cell-surface receptors. Consequently, differences in extraction, purification, and solubility may partly explain apparently inconsistent biological activities reported across studies.

For *S. aspratus*, water-soluble polysaccharide fractions have been associated with macrophage stimulation, including increased nitric oxide production and changes in immune signaling [5]. These observations illustrate why the biological activity of a mushroom polysaccharide should be interpreted together with its structural characteristics and preparation method rather than by β-glucan content alone.

## 4. Health Promoting Effects

Increased evidence of the many health-promoting qualities of *S*. *aspratus* has been a major factor in the increased interest in the plant among scientists. Experimental models have shown that bioactive components, especially polysaccharides and sterol derivatives, have anti-inflammatory, immunomodulatory, anti-cancer, and metabolic regulating properties. These results offer a mechanistic foundation for the historic use of this mushroom as a functional food, even if most of the data comes from in vitro and animal research.

### 4.1. Antioxidant Activity

A major contributing factor to the emergence of chronic illnesses such as diabetes, cardiovascular disease, and cancer is oxidative stress. In common in vitro tests such ferric reducing antioxidant power (FRAP) assays, DPPH radical scavenging, and ABTS radical cation decolorization, extracts and pure polysaccharides from *S. aspratus* have shown a strong antioxidant potential [14]. These assays measure the electron-donating and radical-neutralizing ability of bioactive compounds, and the results indicate that *S. aspratus* polysaccharides possess considerable reducing and free radical scavenging activities.

Mechanistically, the hydroxyl-rich structures of *S. aspratus* polysaccharides are responsible for their antioxidant activity because they promote metal ion chelation and hydrogen donation, which reduce the production of reactive oxygen species (ROS). Furthermore, cellular research indicates that by boosting the activity of enzymes like catalase (CAT) and superoxide dismutase (SOD), these polysaccharides may improve endogenous antioxidant defense mechanisms [5]. These combined mechanisms support the role of *S. aspratus* as a natural antioxidant source.

### 4.2. Anti-Inflammatory Effects

Experimental studies indicate that *S. aspratus* polysaccharides can modulate inflammatory signaling, particularly pathways involving nuclear factor-kappa B (NF-κB), a transcription factor that regulates many inflammatory genes. In experimental systems, *S. aspratus* polysaccharides have been associated with reduced NF-κB activation and lower expression of inflammatory mediators such as cyclooxygenase-2 (COX-2) and inducible nitric oxide synthase (iNOS) [5]. These findings provide a mechanistic link between polysaccharide–receptor interactions and downstream control of inflammatory gene expression.

In immune-cell models, *S. aspratus* extracts have also been reported to alter cytokine production, including decreases in pro-inflammatory cytokines such as interleukin-6 (IL-6) and tumor necrosis factor-alpha (TNF-α), together with increased interleukin-10 (IL-10) under the reported experimental conditions [5]. The direction and magnitude of these effects may depend on the polysaccharide fraction, dose, cell model, and extraction procedure. Therefore, these findings support immunoregulatory potential but do not yet establish a clinically relevant anti-inflammatory effect in humans.

### 4.3. Immunomodulatory Activity

β-Glucan-rich polysaccharide fractions of *S. aspratus* have been associated with immunomodulatory activity in experimental models. Reported responses include increased macrophage activity, phagocytic capacity, nitric oxide production, and immune mediator secretion [5,14]. These effects are consistent with activation of innate immune mechanisms, although the specific contribution of individual *S. aspratus* polysaccharide structures remains incompletely resolved.

**Figure 2:**
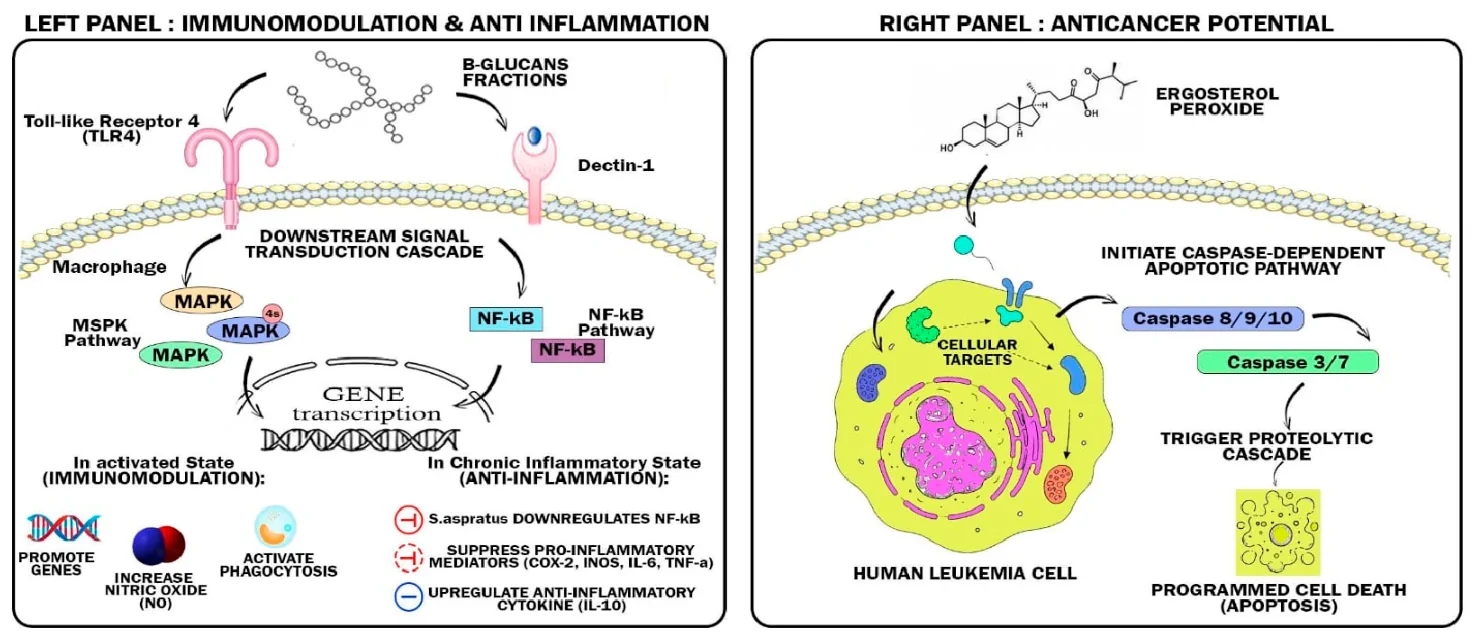
Proposed molecular pathway of S. aspratus bioactivities. The schematic integrates mechanisms supported directly by *S. aspratus* studies with pathways inferred from established mushroom β-glucan and related bioactive-compound research. It is a proposed mechanistic model and has not been experimentally validated as a single integrated pathway in *S. aspratus.*

At the molecular level, β-glucans can interact with pattern-recognition receptors on macrophages and other immune cells, including Dectin-1 and Toll-like receptors. Receptor engagement can activate downstream Syk-, MAPK-, and NF-κB-related signaling and thereby alter cytokine production and macrophage function [5,15]. Figure 2 summarizes these proposed relationships for *S. aspratus*. Because much of the mechanistic evidence is derived from general mushroom β-glucan research, the figure should be interpreted as a proposed model rather than as a fully validated pathway specific to *S. aspratus*.

### 4.4. Anticancer Potential

Ergosterol peroxide is a secondary metabolite identified in *S. aspratus* that has been investigated for anticancer activity. In human leukemia cells, ergosterol peroxide induced apoptosis through caspase-dependent mechanisms [12]. Evidence has also been reported for activity against a solid-tumor cell model: a polysaccharide isolated from *S. aspratus* was reported to trigger apoptosis in human cervical cancer (HeLa) cells through mitochondrial dysfunction [19]. Thus, experimental evidence extends beyond leukemia cells; however, these findings are predominantly in vitro and involve different *S. aspratus* constituents and models. They should therefore not be interpreted as evidence of clinical anticancer efficacy.

The available evidence for anticancer activity remains preliminary. Although *S. aspratus* polysaccharides and ergosterol peroxide have shown apoptosis-related effects in cultured cancer cells, robust in vivo studies and human clinical trials are still lacking. In particular, the mechanisms and potency of individual compounds or polysaccharide fractions may differ substantially among cell types and preparations. Further studies using standardized fractions, appropriate animal models, pharmacokinetic assessment, and eventually clinical trials are required before therapeutic claims can be made.

### 4.5. Metabolic Regulation

Additionally, recent research has shown that *S. aspratus* polysaccharides have metabolism-regulating effects, especially when it comes to obesity-related illnesses. *S. aspratus* polysaccharide supplementation enhanced glucose tolerance, decreased insulin resistance, and altered the makeup of the gut microbiota in mice models fed a high-fat diet [18]. These findings suggest potential anti-diabetic effects mediated through both metabolic and microbiome-related mechanisms.

There have also been reports of lipid-lowering effects in addition to glycemic control. After *S. apratus* polysaccharides are administered, experimental studies show decreases in serum total cholesterol, triglycerides, and hepatic lipid buildup [18]. These results highlight the potential of this mushroom as a functional food ingredient targeting metabolic syndrome and dyslipidemia.

## 5. Mechanistic Insights

To assess the therapeutic potential of *S. aspratus*, it is necessary to comprehend the molecular mechanisms behind its biological activities. According to recent research, this mushroom’s bioactive substances, including polysaccharides and sterol derivatives, may have health-promoting benefits through a variety of cellular processes, including metabolic signaling, oxidative stress management, and immunological regulation.

### 5.1. Molecular Pathways and Signal Transduction

*S. aspratus* polysaccharides may influence intracellular signaling through interactions with pattern-recognition receptors on immune cells. β-Glucan recognition can involve Dectin-1 and, depending on the preparation and cell system, Toll-like receptors, leading to downstream activation of signaling pathways such as Syk, MAPK, and NF-κB and subsequent changes in immune-regulatory gene transcription [5,15]. In parallel, experimental studies of ergosterol peroxide have reported apoptosis-related signaling in leukemia cells, while *S. aspratus* polysaccharides have also induced apoptosis in HeLa cells [12,19]. Figure 2 integrates these findings into a proposed mechanistic framework. Importantly, the complete pathway shown in Figure 2 has not been experimentally validated as a single integrated pathway in *S. aspratus*. Some links are supported directly by *S. aspratus* studies, whereas others are inferred from established mechanisms of mushroom β-glucans or related compounds. The figure should therefore be regarded as a working model that identifies testable mechanistic relationships.

Figure 2 should therefore be read as a synthesis of the available evidence rather than as a direct experimental map of all signaling events in *S. aspratus*. Direct evidence includes the reported effects of *S. aspratus* polysaccharides on immune and metabolic outcomes and the apoptosis-related activity of ergosterol peroxide and *S. aspratus* polysaccharides. Other receptor-to-signaling connections are based on established β-glucan biology and require direct validation in *S. aspratus*.

### 5.2. Oxidative Stress Regulation

An imbalance between the generation of reactive oxygen species (ROS) and antioxidant defenses leads to oxidative stress. By boosting natural antioxidant enzymes including glutathione peroxidase (GPx), catalase (CAT), and superoxide dismutase (SOD), polysaccharides from *S. aspratus* have been demonstrated to lessen oxidative stress. In order to neutralize ROS and stop cellular damage, these enzymes are essential [14]. In addition, the hydroxyl groups present in polysaccharide structures can directly scavenge free radicals, contributing to their antioxidant properties.

### 5.3. Gut Microbiota Modulation

Experimental evidence indicates that *S. aspratus* polysaccharides can influence gut microbiota composition and metabolic parameters in mice fed a high-fat diet [18]. The reported changes in microbial communities were accompanied by improved glucose tolerance, reduced insulin resistance, and altered lipid metabolism. These findings suggest a possible microbiota-mediated contribution to the metabolic effects of the polysaccharides. However, the extent to which these effects result from direct prebiotic activity, altered intestinal fermentation, or other host–microbe interactions remains to be established, and translation to humans requires clinical investigation.

## 6. Safety and Toxicology

The pharmacological characteristics of *S. aspratus* are gaining attention, but little is known about their safety profile. However, the research that is currently available indicates that, when eaten in moderation, edible mushrooms often have low toxicity.

### 6.1. Toxicity Data

Although there aren’t many toxicological investigations on *S. aspratus* at the moment, experimental results show that mushroom polysaccharides have little cytotoxicity against normal cells while retaining biological activity against pathogenic or malignant cells [5]. Furthermore, in experimental settings, sterol derivatives such as ergosterol peroxide have shown specific cytotoxic actions against cancer cells without seriously harming healthy tissues [12].

### 6.2. Safe Dosage Ranges

Studies on the metabolic effects of *S. aspratus* polysaccharides in animals have typically employed doses between 100 and 400 mg/kg of body weight with no discernible side effects [18]. These results suggest a relatively wide safety margin for dietary consumption. Nevertheless, precise dosage recommendations for human consumption remain uncertain due to the absence of clinical studies.

### 6.3. Knowledge Gaps

Comprehensive toxicological evaluations, including long-term safety studies, nontoxicity assessment, and clinical investigations, are still necessary even though preliminary results point to minimal toxicity. To ensure that *S. aspratus* extracts are safe to use as functional food ingredients or in nutraceutical products, such studies are required.

## 7. Current Limitations and Research Gaps

Despite promising findings, several limitations hinder the translation of *S. aspratus* research into clinical applications.

### 7.1. Lack of Clinical Trials

The majority of research on *S. aspratus* has been done in animal models or in vitro. Human clinical studies are required to confirm the medicinal efficacy and safety of substances obtained from mushrooms under real-world circumstances, even though these experiments offer insightful molecular information.

### 7.2. Standardization Issues

The absence of standardized extraction and purification procedures remains a major obstacle to comparing studies. Differences in hot-water extraction, ethanol precipitation, enzymatic treatment, extraction time, temperature, and downstream purification can alter polysaccharide yield, molecular-weight distribution, branching, purity, and biological activity. As a practical framework for future studies, we propose a standardized workflow consisting of ultrasound-assisted hot-water extraction, followed by graded ethanol precipitation and membrane ultrafiltration using defined molecular-weight cut-off (MWCO) membranes. The resulting fractions should then be characterized using standardized measurements of yield, total carbohydrate and β-glucan contents, molecular-weight distribution, monosaccharide composition, glycosidic linkage pattern, purity, and solubility before biological testing. Such a workflow would allow researchers to compare discrete high- and low-molecular-weight fractions more consistently across laboratories and would improve reproducibility during functional-food development.

### 7.3. Bioavailability Concerns

Another crucial factor is bioavailability. The therapeutic efficacy of high-molecular-weight polysaccharides may be impacted by their low absorption in the gastrointestinal tract. To maximize these chemicals’ functional applications, it is consequently essential to comprehend their digestion, metabolism, and systemic distribution.

## 8. Future Perspectives

Given its diverse bioactive compounds and promising pharmacological properties, *S. aspratus* has significant potential for future applications in functional food and nutraceutical development.

### 8.1. Functional Food Applications

As an edible mushroom rich in polysaccharides and bioactive metabolites, *S. aspratus* could be incorporated into functional foods designed to promote immune health, reduce oxidative stress, and support metabolic balance. Developing value-added food products such as fortified beverages, dietary supplements, or mushroom powders may enhance its commercial utilization.

### 8.2. Nutraceutical Development

The isolation and characterization of bioactive compounds from *S. aspratus* also present opportunities for nutraceutical development. Standardized extracts containing polysaccharides or sterol derivatives could be formulated into dietary supplements targeting specific health conditions, including inflammation, metabolic disorders, and immune dysfunction.

### 8.3. Need for Omics-Based Approaches

Advances in modern biotechnology provide new tools for investigating the molecular basis of mushroom bioactivity. Integrative approaches such as genomics, proteomics, metabolomics, and transcriptomics could provide deeper insights into the biosynthesis of bioactive compounds and their mechanisms of action. Such omics-based studies would facilitate the discovery of novel metabolites and molecular targets.

### 8.4. Sustainable Cultivation

Finally, sustainable cultivation strategies are essential to ensure the long-term availability of *S. aspratus*. Developing controlled cultivation systems and optimizing environmental conditions for mushroom production would help reduce dependence on wild harvesting while supporting large-scale commercial applications.

## Figures and Tables

**Table 1:** Phytochemical profile and bioactive constituents of *Sarcodon aspratus*.

Category	Chemical Constituent/Fraction	Key Characteristics & Structural Features	Reported Quantities/Metrics	Biological Relevance & Remarks
Primary Metabolites	Proteins & Amino Acids	Balanced composition of essential and non-essential amino acids.	Essential: 98.88 mg/g Non-essential: 65.49 mg/g.	High nutritional quality and dietary value.
	Water-Soluble Polysaccharides (e.g., HBP, HCP fractions)	Hetero-polysaccharides with beta-linked glucopyranosyl residues.	High molecular weights ranging between ~4.3 × 10^5^ Da and ~6.7 × 10^5^ Da (about 430–670 kDa).	Robust immunomodulatory, antioxidant, and macrophage-activating capacities.
	Fatty Acids	Predominantly unsaturated fatty acids (profile inferred from closely related Basidiomycetes).	Linoleic acid (C18:2) and oleic acid (C18:1) typically predominate.	Standard components of mushroom lipid profiles identified via GC-MS.
	Minerals & Trace Elements	Essential dietary trace elements captured via mineral analysis.	Mg: 751.55 mg/kg Fe: 318.07 mg/kg Zn: 116.77 mg/kg Mn: 33.76 mg/kg.	Confirms the species’ role as a nutrient-dense mineral source.
Secondary Metabolites	Sterols (Ergosterol peroxide)	Lipophilic steroid derivative isolated and confirmed via NMR and MS methods.	Characterized in lipophilic extracts.	Demonstrates potent, selective apoptosis induction in human leukemia cells.
	Terpenoids & p-Terphenyls	Cyathane diterpenoids, p-terphenyl derivatives, and non-volatile natural compounds.	Isolated using chromatographic separation and preparative HPLC.	Associated with general antioxidant and antibacterial activities across the *Sarcodon* genus.
	Cerebrosides	Rare, lipid-derived sphingoid metabolites.	Newly discovered via preparative HPLC and purification.	Illustrates the unique chemical distinctness and structural diversity of the species.
	Small Molecules (l-pipecolic acid)	Small molecule enzyme inhibitor.	Extracted and purified from the mushroom.	Exhibits in vitro suppression of angiotensin I-converting enzyme (potential antihypertensive benefits).

## Data Availability

No new data were created or analyzed in this study. Data sharing is not applicable to this article.
